# Quantitative effects of sodium–glucose cotransporter-2 inhibitors dapagliflozin and empagliflozin on quality of life in heart failure patients

**DOI:** 10.3389/fphar.2022.910858

**Published:** 2022-11-28

**Authors:** Dong-Dong Wang, Cun Zhang, Ping Zhu, Su-Mei He, Xiao Chen

**Affiliations:** ^1^ Jiangsu Key Laboratory of New Drug Research and Clinical Pharmacy and School of Pharmacy, Xuzhou Medical University, Xuzhou, China; ^2^ Department of Pharmacy, Xuzhou Oriental Hospital Affiliated to Xuzhou Medical University, Xuzhou, China; ^3^ Department of Endocrinology, Huaian Hospital of Huaian City, Huaian, China; ^4^ Department of Pharmacy, Suzhou Science and Technology Town Hospital, Suzhou, China; ^5^ School of Nursing, Xuzhou Medical University, Xuzhou, China

**Keywords:** Kansas City Cardiomyopathy Questionnaire, dapagliflozin, empagliflozin, heart failure patients, nonlinear mixed-effect modeling

## Abstract

The aim of the present study is to investigate the quantitative effects of sodium–glucose cotransporter-2 (SGLT-2) inhibitors on the quality of life in heart failure (HF) patients. A total of 14,674 HF patients from two dapagliflozin and three empagliflozin studies is included for analysis *via* the nonlinear mixed-effect modeling (NONMEM) software, among which the change rate of the Kansas City Cardiomyopathy Questionnaire (KCCQ) score is used as the evaluation index. There is no significant difference in the pharmacodynamics influencing the quality of life in HF patients between the SGLT-2 inhibitors: 10 mg/day dapagliflozin and 10 mg/day empagliflozin. For the clinical summary score (CSS), total symptom score (TSS), and overall summary score (OSS), the E_max_ of the SGLT-2 inhibitors on the quality of life in HF patients is 3.74%, 4.43%, and 4.84%, respectively, and ET_50_ is 2.23, 4.37, and 7.15 weeks, respectively. In addition, the time duration of achieving 25%, 50%, 75%, and 80% E_max_ is 0.75, 2.23, 6.69, and 8.92 weeks for the CSS; 1.46, 4.37, 13.11, and 17.48 weeks for the TSS; and 2.39, 7.15, 21.45, and 28.6 weeks for the OSS, respectively. Therefore, to reach the plateau period (80% of E_max_) of SGLT-2 inhibitors on the CSS, TSS, and OSS, 10 mg/day dapagliflozin (or 10 mg/day empagliflozin) is required to be taken for 8.92 weeks, 17.48 weeks, and 28.6 weeks, respectively. This is the first time that the quantitative effects of SGLT-2 inhibitors on the quality of life in HF patients are being explored.

## Introduction

Heart failure (HF) is a functionally abnormal heart on account of either structural and/or functional deficits inducing an elevation in intracardiac pressures and/or reduced cardiac output, which has become a growing medical problem (Badoer, 2022). Recent data indicate that the incidence of HF is increasing year by year (Roth et al., 2015; Conrad et al., 2018; Cleland et al., 2019). At the same time, it has brought a huge economic burden on society (Bozkurt et al., 2021). Based on world standards, China and India are the two countries with the fastest rising rates of HF (Bragazzi et al., 2021). Combined with the large populations of these two countries, the HF problem in future will be even more serious (Badoer, 2022). Therefore, more efforts are required to be put into the prevention and treatment of HF patients.

Sodium–glucose cotransporter-2 (SGLT-2) inhibitors are a group of antidiabetic drugs, which are located in the S1 segment of the renal proximal tubule and account for the absorption of nearly 90% of the glucose by the kidneys (Turner and Moran, 1982; Kashiwagi and Maegawa, 2017). In addition to lowering blood glucose, SGLT-2 inhibitors also have the power to reduce the risk of cardiovascular outcomes and mortality (Mascolo et al., 2021), especially when HF patients can obtain benefits from therapy treatment by dapagliflozin and empagliflozin (Packer et al., 2020; Butt et al., 2021). However, the quantitative effects of SGLT-2 inhibitors dapagliflozin and empagliflozin on the quality of life in HF patients are still unknown. The present study aims to explore the quantitative effects of SGLT-2 inhibitors on the quality of life in HF patients.

## Methods

### Enrolled patients

A total of 14,674 HF patients treated by SGLT-2 inhibitors, namely, dapagliflozin and empagliflozin, from published literature have been included for analysis (Kosiborod et al., 2020; Abraham et al., 2021; Butler et al., 2021; Nassif et al., 2021; Butler et al., 2022a). The strategy used for literature search is given in the Supplementary Material. The source, group, dosage, duration of treatment, number of people, age, clinical summary score (CSS), total symptom score (TSS), and overall summary score (OSS) have been collected from the published literature included for analysis.

For eliminating the potential baseline effect, the change rate of the KCCQ score is used as the evaluation index, which includes the CSS, TSS, and OSS. Eq. 1 describes the calculation method:

$$S_{k}=\frac{S_{k,time}-S_{k,base}}{S_{k,base}}\times 100\%.$$ (1)

where k represents the KCCQ score which includes the CSS, TSS, and OSS; S_k_ represents the change rate of the KCCQ score; S_k,time_ represents the value of the KCCQ score at a time; S_k,base_ represents the value of the KCCQ score at baseline.

### Model building

The quantitative effects of the SGLT-2 inhibitors on the KCCQ in HF patients are evaluated by the E_max_ model. For obtaining the actual effects of the SGLT-2 inhibitors on the KCCQ in HF patients, the placebo control group effects are subtracted from the sum effects, which are shown in Eqs 2, 3:

$$S_{a,k,i,j}{=S}_{s,k,i,j}{-S}_{p,k,i,j}$$ (2)

$$S_{a,k,i,j}=\frac{E_{\max,k,i,j}\times Time}{{ET}_{50,k,i,j}+Time}+\frac{\varepsilon_{k,i,j}}{\sqrt{\frac{N_{k,i,j}}{1000}}}.$$ (3)

where S_s,k,i,j_ represents the sum effects of the SGLT-2 inhibitors on the KCCQ in HF patients; S_p,k,i,j_ represents the placebo control group effects on the KCCQ in HF patients; S_a,k,i,j_ represents the actual effects of the SGLT-2 inhibitors on the KCCQ in HF patients; k represents the KCCQ score, which includes CSS, TSS, and OSS; i represents different studies; and j represents the time point. E_max,k_ represents the maximal effects on the KCCQ; ET_50,k_ represents the duration of treatment to reach half of the maximal effects; Ɛ_k,i,j_ represents the residual error of study i with j time under different KCCQ scores, which include the CSS, TSS, and OSS; N_k,i,j_ represents the sample size in study i with time point j under different KCCQ scores, which include the CSS, TSS, and OSS; Ɛ_k,i,j_ is weighted by the sample size, assumed to be normally distributed, with a mean of 0 and variance of σ^2^/(N_k,i,j_/1,000).

The variability of the inter-study is described by the additive or exponential error model, given by Eqs 4–7:

$$E_{\max,k,i,j}=E_{\max,k}+\eta_{k,1,i}$$ (4)

$${ET}_{50,k,i,j}={ET}_{50,k}+\eta_{k,2,i}$$ (5)

$$E_{\max,k,i,j}=E_{\max,k}\times \exp\left(\eta_{k,1,i}\right)$$ (6)

$${ET}_{50,k,i,j}={ET}_{50,k}\times \exp\left(\eta_{k,2,i}\right).$$ (7)

η_k,1,i_ and η_k,2,i_ represent the inter-study variabilities, which are assumed to be normally distributed, with a mean of 0 and variance of ω_k,1,i_^2^ and ω_k,2,i_^2^, when available, they would be added into E_max, k_ or ET_50, k_, respectively; k represents the KCCQ score, which includes the CSS, TSS, and OSS.

Furthermore, Eqs 8–10 describe continuous or categorical covariates:

$$S_{i}=S_{T}+\left(COV-{COV}_{m}\right)\cdot \theta_{c}$$ (8)

$$S_{i}=S_{T}\times {\left(COV/{COV}_{m}\right)}^{\theta c}$$ (9)

$$S_{i}=S_{T}+COV\times \theta_{c}.$$ (10)

where S_i_ represents the parameter for a patient with a covariate value of COV; S_T_ represents the typical value of the parameter; COV represents the covariate; COV_m_ represents the median value of the covariable in the population. θ_c_ represents a correction coefficient of the covariate to the model parameter. Dapagliflozin and empagliflozin are also selected as potential covariables to evaluate whether there is a significant difference in the pharmacodynamics between the two drugs.

The study is conducted *via* the nonlinear mixed-effect modeling (NONMEM, edition 7, ICON Development Solutions, Ellicott City, MD, United States) software. After the basic model is set up, the potential covariates are considered for adding into E_max,k_ or ET_50,k_. The objective function value (OFV) change is selected as the covariate inclusion criterion, where if the OFV decreases more than 3.84 (χ^2^, α *=* 0.05, d.f. = 1), it is considered sufficient for inclusion; however, if the OFV increases more than 6.63 (χ^2^, α *=* 0.01, d.f. = 1), it is considered sufficient for significance in the final model (Wang et al., 2021).

### Model evaluation

The final models are evaluated by the observations *vs.* individual predictions and conditional weighted residuals (WRES) *vs.* individual predictions *vs*. individual plots. The predictive performance of the final models is assessed by visual predictive check (VPC) plots.

### Prediction

The curves of the final models from the CSS, TSS, and OSS are simulated using the Monte Carlo method, which includes the time required to achieve 25%, 50%, 75%, and 80% E_max_ of the SGLT-2 inhibitors on the KCCQ in HF patients.

## Results

### Included patients

HF patients from two dapagliflozin and three empagliflozin studies are included for analysis (Kosiborod et al., 2020; Abraham et al., 2021; Butler et al., 2021; Nassif et al., 2021; Butler et al., 2022a). The dosages of dapagliflozin and empagliflozin are 10 mg/day. The KCCQ score of HF patients include the CSS, TSS, and OSS. The detailed information is given in Table 1.

**TABLE 1:** Studies identified for analysis.

Studies	Source	Group	Dosage	Duration of treatment	Number of people	Age (years)	Clinical summary score	Total symptom score	Overall summary score
Nassif et al. (2021)	United States	Dapagliflozin	10 mg/day	12 weeks	162	69 (64 and 77)	63.4 ± 19.7	—	63.2 ± 20.4
		Placebo	—	12 weeks	162	71 (63 and 78)	61.8 ± 20.3	—	62.3 ± 20.6
Kosiborod et al. (2020)	Multinational	Dapagliflozin	10 mg/day	8 months	2,222	66.2 ± 11.0	71.0	73.80	68.30
		Placebo	—	8 months	2,221	66.5 ± 10.8	71.3	73.90	68.70
Butler et al. (2022a)	Multinational	Empagliflozin	10 mg/day	52 weeks	2,884	71.9	70.4 (21.2)	73.5 (22.0)	68.9 (21.1)
		Placebo	—	52 weeks	2,867	71.9	70.4 (21.2)	73.5 (22.0)	68.9 (21.1)
Butler et al. (2021**)**	Multinational	Empagliflozin	10 mg/day	12 months	1,776	66.9	70.7 (21.9)	—	—
		Placebo	—	12 months	1,753	66.9	70.7 (21.9)	—	—
Abraham et al. (2021**)**	Multinational	Empagliflozin 1	10 mg/day	12 weeks	156	69.0 (62.5 and 77.0)	—	68.8 (50.5 and 83.3)	—
		Placebo 1	—	12 weeks	156	70.0 (62.5 and 77.0)	—	68.8 (49.5 and 87.5)	—
		Empagliflozin 2	10 mg/day	12 weeks	157	74.0 (68.0 and 79.0)	—	64.6 (46.9 and 84.4)	—
		Placebo 2	—	12 weeks	158	75.0 (68.0 and 81.0)	—	68.2 (49.0 and 86.5)	—

### Modeling

The parameter estimates of the final model are shown in Table 2. For the CSS, TSS, and OSS, the E_max_ of the SGLT-2 inhibitors on the quality of life in HF patients is 3.74%, 4.43%, and 4.84%, respectively, and the ET_50_ is 2.23 weeks, 4.37 weeks, and 7.15 weeks, respectively. In addition, the different SGLT-2 inhibitors, namely, dapagliflozin or empagliflozin, are not the covariates included in the final model, showing that there is no significant difference in the pharmacodynamics influencing the quality of life in HF patients between the SGLT-2 inhibitors: 10 mg/day dapagliflozin and 10 mg/day empagliflozin from the studies so far included in the analysis.

**TABLE 2:** Parameter estimates of the final model and 90% confidence interval.

Model	Parameter	Estimate	Simulation	
			Median	90% confidence interval
CSS	E_max_, %	3.74	3.74	[2.29 and 6.89]
	ET_50_, week	2.23	2.23	[0.52 and 13.60]
	ω_Emax_	0.525	0.452	[0.003 and 0.675]
	ω_ET50_	1.881	0.773	[0.003 and 3.435]
	Ɛ	0.185	0.188	[0.045 and 0.308]
TSS	E_max_, %	4.43	4.01	[3.01 and 6.85]
	ET_50_, week	4.37	4.37	[0.52 and 8.48]
	ω_Emax_	0.332	0.287	[0.113 and 0.396]
	ω_ET50_	—	—	—
	Ɛ	0.146	0.146	[0.010 and 0.175]
OSS	E_max_, %	4.84	4.81	[3.28 and 5.81]
	ET_50_, week	7.15	7.15	[6.54 and 8.17]
	ω_Emax_	0.483	0.289	[0.003 and 0.575]
	ω_ET50_	0.079	0.003	[0.003 and 0.079]
	Ɛ	0.020	0.020	[0.010 and 0.024]

CSS is the clinical summary score; TSS is the total symptom score; OSS is the overall summary score; 90% confidential interval is shown with the 5th and 95th percentile; E_max_ is the maximal effect; ET_50_ is the treatment duration to reach half of E_max_; ω_Emax_ is the inter-study variability of E_max_; ω_ET50_ is the inter-study variability of ET_50_; Ɛ is the residual error.

Finally, the relationships between the SGLT-2 inhibitors (dapagliflozin and empagliflozin) and the quality of life in HF patients are shown in Eqs. 11–13:

$$S=\frac{-3.74\%\times Time}{2.23+Time}$$ (11)

$$S=\frac{-4.43\%\times Time}{4.37+Time}$$ (12)

$$S=\frac{-4.84\%\times Time}{7.15+Time}.$$ (13)

where S represents the change rate of the KCCQ score, and Eqs. 11–13 are the CSS, TSS, and OSS, respectively. Time is the duration of treatment for the SGLT-2 inhibitors in HF patients.

### Evaluation

**FIGURE 1:**
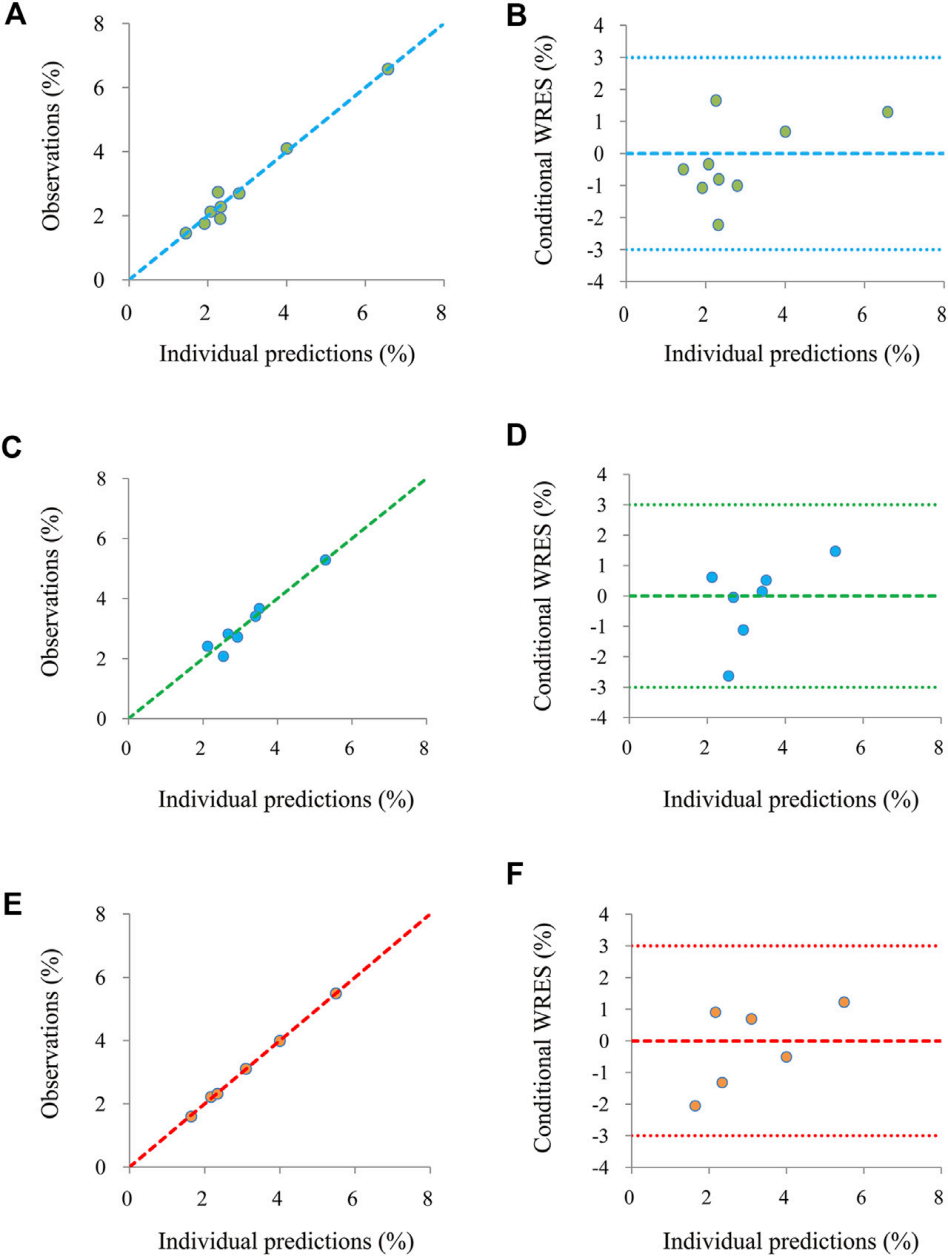
Model evaluation. **(A,B)** were from the CSS, **(C,D)** were from the TSS, and **(E,F)** were from the OSS. CSS: clinical summary score; TSS: total symptom score; OSS: overall summary score.

**FIGURE 2:**
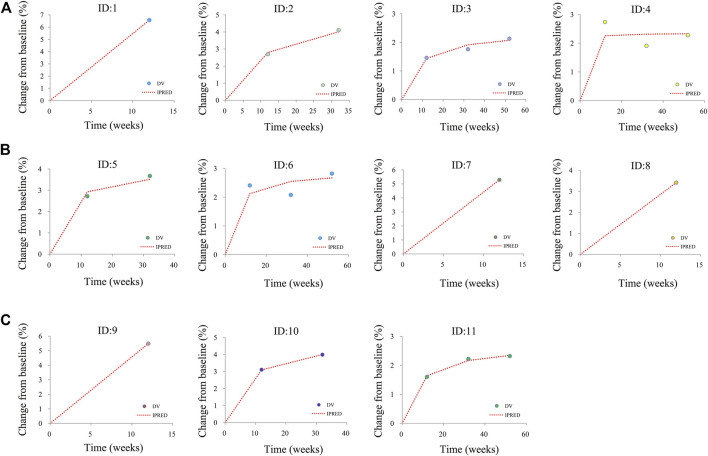
Individual plots. **(A)** was from CSS, **(B)** was from TSS, and **(C)** was from OSS; ID: 1–11 were from the studies (Kosiborod et al., 2020; Abraham et al., 2021; Butler et al., 2021; Nassif et al., 2021; Butler et al., 2022a). CSS: clinical summary score; TSS: total symptom score; OSS: overall summary score.

**FIGURE 3:**
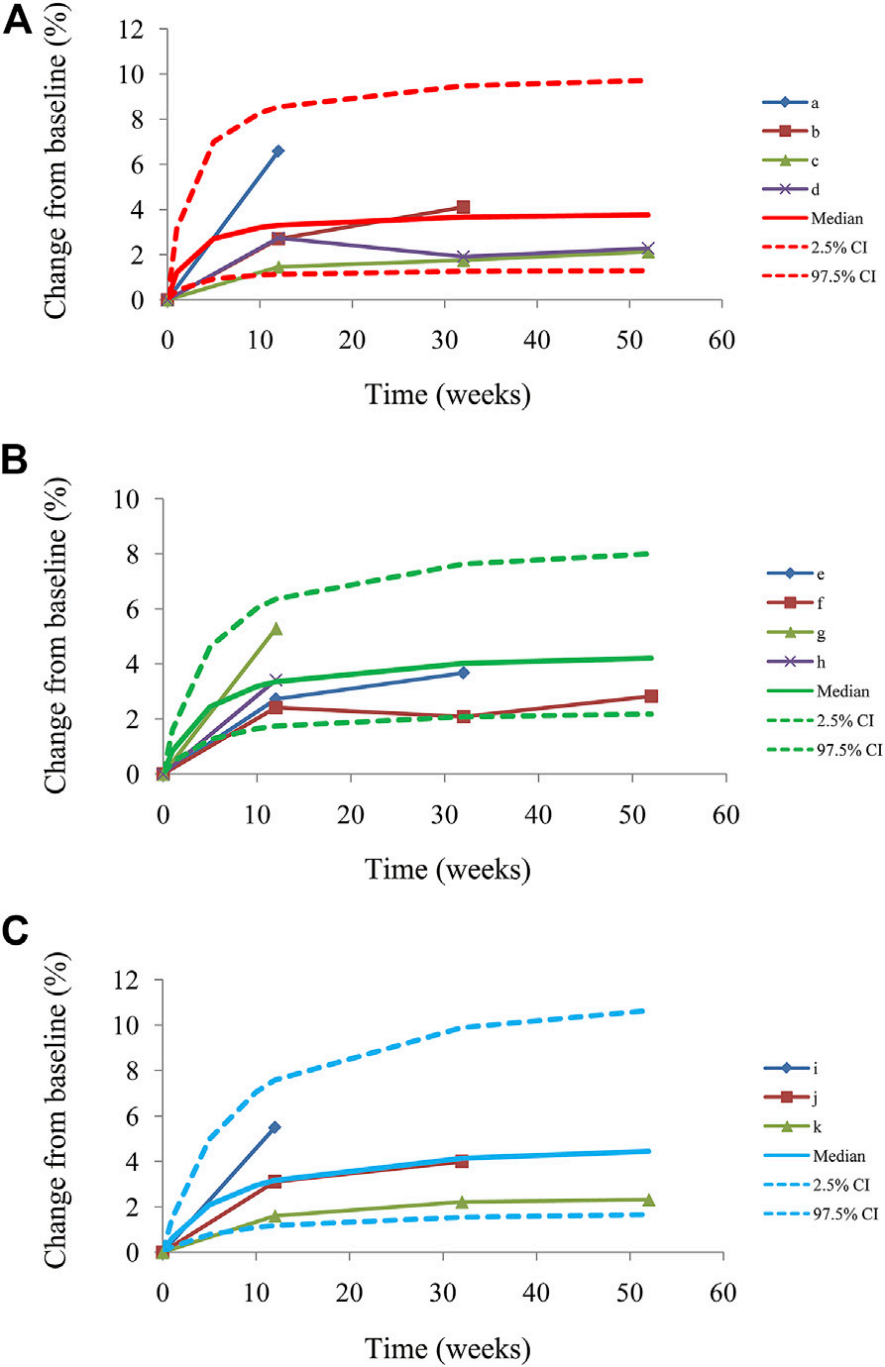
Visual predictive check plots from CSS **(A)**, from TSS **(B)**, from OSS **(C)**, and a–k from the studies (Kosiborod et al., 2020; Abraham et al., 2021; Butler et al., 2021; Nassif et al., 2021; Butler et al., 2022a). Median, 2.5% CI and 97.5% CI were simulated by the Monte Carlo method (n = 1,000); CI, confidence interval. CSS: clinical summary score; TSS: total symptom score; OSS: overall summary score.

Figure 1 shows the goodness-of-fit plots of the model, which include observations *vs.* individual predictions and conditional weighted residuals (WRES) *vs.* individual plots. Figures 1A,B are from the CSS model, Figures 1C,D are from the TSS model, and Figures 1E,F are from the OSS model. They show good linear relationships between individual predictions and observations, indicating the better fitting of the final models. Figure 2 shows individual plots, Figures 2A–C are from the CSS, TSS, and OSS, respectively. They demonstrate acceptable predictability from the perspective of the clinical sparse data. Figure 3 shows the VPC plots, and Figures 3A–C are from the CSS, TSS, and OSS, respectively. They indicate the most observed data that are included in the 95% prediction intervals produced with the simulation data, meaning the predictive power of the final models.

### Prediction

**FIGURE 4:**
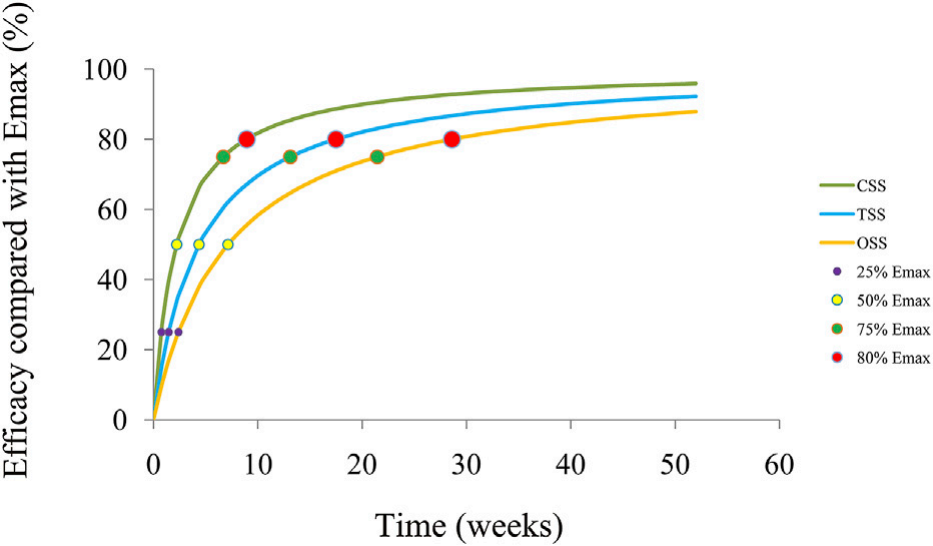
Model prediction. CSS: clinical summary score; TSS: total symptom score; OSS: overall summary score.

The trends of efficacy of the SGLT-2 inhibitors on the quality of life in HF patients are shown in Figure 4. The time duration to achieve 25%, 50%, 75%, and 80% E_max_ is 0.75, 2.23, 6.69, and 8.92 weeks for the CSS; 1.46, 4.37, 13.11, and 17.48 weeks for the TSS; and 2.39, 7.15, 21.45, and 28.6 weeks for the OSS, respectively. Therefore, to reach the plateau period (80% of E_max_) of the SGLT-2 inhibitors on the CSS, TSS, and OSS, 10 mg/day dapagliflozin (or 10 mg/day empagliflozin) is required to be taken for 8.92, 17.48, and 28.6 weeks, respectively.

## Discussion

HF is a syndrome of complex diseases, which could lead to death in severe cases and bring some challenges to treatment and nursing (Fiuzat et al., 2020; Wang A. et al., 2022). For patients with HF, the treatment objectives are to alleviate clinical symptoms, delay disease progression, improve long-term prognosis, reduce mortality and hospitalization rates, and improve the quality of life in patients as much as possible. In general, patients with HF need to take comprehensive treatment, which mainly includes removing the inducement, etiology, and symptomatic support treatments such as surgery and drug therapy, among which drug therapy is one of the most common means (Wang Y. et al., 2022).

SGLT-2 inhibitors are a newer group of antidiabetic drugs, with novel and unexpected pleiotropic effect. However, the number of relevant studies is increasing and the evidence of benefit on HF, and patients' quality of life, is mounting. Research in recent years has found that SGLT-2 inhibitors have the power to reduce the risk of cardiovascular outcomes and mortality (Mascolo et al., 2021), especially on HF patients who could gain benefits from therapy treatment with dapagliflozin and empagliflozin (Packer et al., 2020; Butt et al., 2021). The mechanism of the benefits of SGLT-2 inhibitors in HF is not clear, but includes improvement in myocardial energetics (Garcia-Ropero et al., 2019) due to enhanced cardiac consumption of fatty acids and ketone bodies (Santos-Gallego et al., 2019), reverse cardiac remodeling (Santos-Gallego et al., 2021b), improvement in diastolic function (Santos-Gallego et al., 2021a), natriuretic and diuretic effects that reduce congestion (Mordi et al., 2020), inhibition in sodium–hydrogen exchanger (Baartscheer et al., 2017), improvement in aortic stiffness (Requena-Ibanez et al., 2021), and systemic vascular resistance (Chilton et al., 2015).

In addition, the EMPA-TROPISM trial also corroborates the improvement in the quality of life in HF patients with SGLT-2 inhibitors (Requena-Ibanez et al., 2022). The cardiopulmonary exercise test (CPET) is a more sensitive technique to evaluate exercise capacity and quality of life. Of utmost importance, the SGLT-2 inhibitors also improve peak VO_2_ (the most sensitive parameter for exercise capacity and quality of life) in HF patients, both empagliflozin (Santos-Gallego et al., 2021b) and dapagliflozin (Palau et al., 2022). However, the quantitative effects of SGLT-2 inhibitors dapagliflozin and empagliflozin on the quality of life in HF patients remain unknown. The present study aims to explore the quantitative effects of SGLT-2 inhibitors on the quality of life in HF patients.

The KCCQ, assessing the influence on HF from the perspective of patients in their health status, is a disease-specific measure item (Green et al., 2000), which has been proved to be sensitive, valid, and reliable to clinical changes and at the same time associated with hospitalization, costs, and death (Spertus and Jones, 2015; Pokharel et al., 2017), where the CSS quantifies the physical function and symptoms domains, TSS includes symptom frequency and severity, OSS is sourced from the TSS, physical function, quality of life, and social function, and these are scored from a range of 0–100, with higher scores reflecting a better health status (Butler et al., 2021). At present, more and more studies have taken the KCCQ as a key evaluation index in the treatment of patients with HF. For example, Wohlfahrt et al. (2022) report HF-related quality-of-life impairment after myocardial infarction. DeVore et al. (2022) report the association of improvement in the left ventricular ejection fraction with outcomes in patients with HF with a reduced ejection fraction: data from CHAMP-HF. Butler et al. (2022b) report health status improvements with ferric carboxymaltose in HF with reduced ejection fraction and iron deficiency. In the abovementioned studies, the KCCQ is introduced as an important clinical evaluation indicator. Therefore, in this study, we choose the KCCQ, which includes the CSS, OSS, and TSS, as the evaluation index to explore the quantitative effects of the SGLT-2 inhibitors on the quality of life in HF patients.

In the present study, a total of 14,674 HF patients from two dapagliflozin and three empagliflozin studies have been included for analysis *via* NONMEM, among which the change rate of the KCCQ, which includes the CSS, OSS, and TSS, are used as the evaluation index. In the final model, there is no significant difference in the pharmacodynamics influencing the quality of life in HF patients between the SGLT-2 inhibitors: 10 mg/day dapagliflozin and 10 mg/day empagliflozin. For the CSS, TSS, and OSS, the E_max_ of the SGLT-2 inhibitors on the quality of life in HF patients is 3.74%, 4.43%, and 4.84%, respectively, and the ET_50_ is 2.23, 4.37, and 7.15 weeks, respectively. In addition, the time duration of achieving 25%, 50%, 75%, and 80% E_max_ is 0.75, 2.23, 6.69, and 8.92 weeks for the CSS; 1.46, 4.37, 13.11, and 17.48 weeks for the TSS; and 2.39, 7.15, 21.45, and 28.6 weeks for the OSS, respectively. Therefore, to reach the plateau period (80% of E_max_) of the SGLT-2 inhibitors on the CSS, TSS, and OSS, 10 mg/day dapagliflozin (or 10 mg/day empagliflozin) is required to be taken for 8.92, 17.48, and 28.6 weeks, respectively.

In addition, to eliminate the potential baseline effect, the change rate of the KCCQ score is used as the evaluation index. To obtain the actual effects of the SGLT-2 inhibitors on the KCCQ in HF patients, the placebo control group effects are subtracted from the sum effects. Of course, there is no difference in the ejection fraction between the experimental group and control group from the same research source. By this data calculation, we can exclude the effect of different ejection fractions on the pharmacodynamics of the SGLT-2 inhibitors. In this way, the actual effects that we obtain of the SGLT-2 inhibitors on the KCCQ in HF patients are simply the effects of the SGLT-2 inhibitors without the effects from the ejection fractions.

However, the present study also has objective limitations. The SGLT-2 inhibitors analyzed in this study mainly include dapagliflozin and empagliflozin because other class drugs have not been reported or the number of their studies is too small to be analyzed. Therefore, more drugs from the SGLT-2 inhibitors and more studies are required to be further analyzed in the future.

## Conclusion

This is the first time that the quantitative effects of the SGLT-2 inhibitors on the quality of life in HF patients have been explored. To reach the plateau period (80% of E_max_) of the SGLT-2 inhibitors on the CSS, TSS, and OSS, 10 mg/day dapagliflozin (or 10 mg/day empagliflozin) is required to be taken for 8.92, 17.48, and 28.6 weeks, respectively.

## Data Availability

The original contributions presented in the study are included in the article/Supplementary Material; further inquiries can be directed to the corresponding authors.
