# Supplementary material for: Quantitative effects of sodium–glucose cotransporter-2 inhibitors dapagliflozin and empagliflozin on quality of life in heart failure patients
Source: Front Pharmacol. 2022 Nov 28;13:910858. doi: 10.3389/fphar.2022.910858 (PMC9742417; doi:10.3389/fphar.2022.910858)
Supplement: Supplementary file 1 [file Table1.DOCX]

**Table 1S. Search details**

| No. | Query | Results |
| --- | --- | --- |
| #1 | Dapagliflozin[Title] | 999 |
| #2 | Empagliflozin[Title] | 931 |
| #3 | KCCQ | 368 |
| #4 | #1 OR #2 | 1,906 |
| #5 | #3 AND #4 | 11 |
| #6 | #10 Filters: Humans | 7 |

**Figure 1S. Strategy for literature search**

**List of final included studies (n=5)**

**Dapagliflozin (n=2)**

**1.** Nassif ME, Windsor SL, Borlaug BA, Kitzman DW, Shah SJ, Tang F, Khariton Y, Malik AO, Khumri T, Umpierrez G, Lamba S, Sharma K, Khan SS, Chandra L, Gordon RA, Ryan JJ, Chaudhry SP, Joseph SM, Chow CH, Kanwar MK, Pursley M, Siraj ES, Lewis GD, Clemson BS, Fong M, Kosiborod MN. The SGLT2 inhibitor dapagliflozin in heart failure with preserved ejection fraction: a multicenter randomized trial. Nat Med. 2021 Nov;27(11):1954-1960. doi: 10.1038/s41591-021-01536-x. Epub 2021 Oct 28. PMID: 34711976; PMCID: PMC8604725.

**2.** Kosiborod MN, Jhund PS, Docherty KF, Diez M, Petrie MC, Verma S, Nicolau JC, Merkely B, Kitakaze M, DeMets DL, Inzucchi SE, Køber L, Martinez FA, Ponikowski P, Sabatine MS, Solomon SD, Bengtsson O, Lindholm D, Niklasson A, Sjöstrand M, Langkilde AM, McMurray JJV. Effects of Dapagliflozin on Symptoms, Function, and Quality of Life in Patients With Heart Failure and Reduced Ejection Fraction: Results From the DAPA-HF Trial. Circulation. 2020 Jan 14;141(2):90-99. doi: 10.1161/CIRCULATIONAHA.119.044138. Epub 2019 Nov 17. PMID: 31736335; PMCID: PMC6964869.

**Empagliflozin (n=3)**

**3.** Butler J, Filippatos G, Jamal Siddiqi T, Brueckmann M, Böhm M, Chopra VK, Pedro Ferreira J, Januzzi JL, Kaul S, Piña IL, Ponikowski P, Shah SJ, Senni M, Vedin O, Verma S, Peil B, Pocock SJ, Zannad F, Packer M, Anker SD. Empagliflozin, Health Status, and Quality of Life in Patients With Heart Failure and Preserved Ejection Fraction: The EMPEROR-Preserved Trial. Circulation. 2022 Jan 18;145(3):184-193. doi: 10.1161/CIRCULATIONAHA.121.057812. Epub 2021 Nov 15. PMID: 34779658; PMCID: PMC8763045.

**4.** Butler J, Anker SD, Filippatos G, Khan MS, Ferreira JP, Pocock SJ, Giannetti N, Januzzi JL, Piña IL, Lam CSP, Ponikowski P, Sattar N, Verma S, Brueckmann M, Jamal W, Vedin O, Peil B, Zeller C, Zannad F, Packer M; EMPEROR-Reduced Trial Committees and Investigators. Empagliflozin and health-related quality of life outcomes in patients with heart failure with reduced ejection fraction: the EMPEROR-Reduced trial. Eur Heart J. 2021 Mar 31;42(13):1203-1212. doi: 10.1093/eurheartj/ehaa1007. PMID: 33420498; PMCID: PMC8014525.

**5.** Abraham WT, Lindenfeld J, Ponikowski P, Agostoni P, Butler J, Desai AS, Filippatos G, Gniot J, Fu M, Gullestad L, Howlett JG, Nicholls SJ, Redon J, Schenkenberger I, Silva-Cardoso J, Störk S, Krzysztof Wranicz J, Savarese G, Brueckmann M, Jamal W, Nordaby M, Peil B, Ritter I, Ustyugova A, Zeller C, Salsali A, Anker SD. Effect of empagliflozin on exercise ability and symptoms in heart failure patients with reduced and preserved ejection fraction, with and without type 2 diabetes. Eur Heart J. 2021 Feb 11;42(6):700-710. doi: 10.1093/eurheartj/ehaa943. PMID: 33351892.
